# Pediatric Anesthesia Specialty Societies and Multi-Institutional Collaborations

**DOI:** 10.3390/children7110233

**Published:** 2020-11-17

**Authors:** Camila B. Walters, James M. Kynes, Srijaya K. Reddy, Christy J. Crockett, Hannah K. Lovejoy, Amanda N. Lorinc

**Affiliations:** Division of Pediatric Anesthesia, Vanderbilt University Medical Center, Nashville, TN 37232, USA; j.matt.kynes@vumc.org (J.M.K.); srijaya.k.reddy@vumc.org (S.K.R.); christy.crockett@vumc.org (C.J.C.); hannah.lovejoy@vumc.org (H.K.L.)

**Keywords:** pediatric anesthesia, international and national collaborations, multi-institution research

## Abstract

Pediatric anesthesiology is a subspecialty of anesthesiology that deals with the high-risk pediatric population. The specialty has made significant advancement in large collaborative efforts to study and increase patient safety, including the creation of international societies, a dedicated journal, special committees and interest groups, and multi-institution databases for research and quality improvement. Readily available resources were created to help with the education of future pediatric anesthesiologists as well as continuing medical education. Conclusions: Specialty societies and collaborations in pediatric anesthesia are crucial for continuous improvement in the care of children. They promote research, education, quality improvement, and advocacy at the local, national, and international level.

## 1. Introduction

Anesthesiology is a leading medical specialty in patient safety with specialty improvement efforts contributing to a reduction in mortality rates attributable to anesthesia from 1:2500 to 1:13,000 in 50 years [1,2,3]. In the United States, an estimated 6 million children (including 1.5 million infants) undergo surgery each year [4]. Children are known for having a higher risk of anesthetic complications in the perioperative period. Therefore, a subspecialty of anesthesiology, pediatric anesthesia, devotes itself to improving patient safety in this vulnerable population. Significant efforts have been made to this end with notable multi-institutional national and international collaborations that allow for exchange of information, large data gathering, development of guidelines, and changes in practice that move the specialty forward. These collaborations are important for continuous improvement in the care of children.

Pediatric anesthesia was born in the early 20th century along with advancements of pediatric surgery by Dr. William Ladd at Children’s Hospital Boston. Advancements in pediatric surgery demanded strong pediatric anesthesia skills. One of the first pediatric anesthesiologists was Dr. Charles Robson who became Chair at the Hospital for Sick Children in Toronto in 1919 [5]. The Ayre T-piece, a pediatric-specific breathing circuit, was developed by Dr. Philip Ayre and described in 1937 [6]. Training and education programs in North America were developed by Dr. Morton Digby Leigh in 1940, with the first textbook dedicated to the specialty in 1948 [7]. Perhaps the most famous pediatric anesthesiologist, Virginia Apgar, developed the APGAR score in 1953 for the assessment of newborns, and it is still widely used [8]. Respiratory distress syndrome had a mortality of greater than 50% in premature neonates when President John F. Kennedy’s son succumbed to this common pathology [5]. The mortality risk decreased rapidly to 2% by 1988 with pediatric critical care advancements [9]. Pediatric intensive care units were formed in the 1950s. In the 1960s and 1970s, advancements in resuscitation, monitoring, and ventilation led to the specialty of pediatric critical care medicine [5,10,11]. Pediatric anesthesia is now a separate subspecialty according to the Accreditation Council of Graduate Medical Education. The subspecialty has a dedicated journal called *Pediatric Anesthesia*, societies in every continent (Table 1), and many collaborative efforts. 

## 2. Pediatric Anesthesia Collaborations

### 2.1. Pediatric Anesthesia Societies

Country-specific and international subspecialty societies have been formed dedicated to pediatric anesthesia with increasing numbers over the years, growing from 13 in 2012 to 20 in 2020 (Table 1) [5,12]. Efforts are underway to develop these associations in countries that do not yet have them, such as Kenya. These societies have generated a myriad of educational efforts and projects including the collaboration between the Association of Anaesthetists of Great Britain and Ireland (APAGBI) and the World Federation of Societies of Anaesthesiologists’ (WFSA) Safer Anaesthesia from Education (SAFE) paediatric anaesthesia course, which is taught in several countries [13]. 

In addition to pediatric anesthesia societies, pediatric surgery groups lobby for safe perioperative care including the delivery of safe anesthesia. These include the Global Initiative for Children’s Surgery [14], KidsOR, and the World Federation of Associations of Pediatric Surgeons. 

### 2.2. Society for Pediatric Anesthesia 

The Society for Pediatric Anesthesia (SPA) was founded in 1986 in the United States to encourage research, education, and scientific progress in pediatric anesthesia. As of September 2020, it has 3541 members. The mission statement of the SPA states that the society advances the safety and quality of anesthesia care, perioperative care, and pain management in children by educating clinicians, supporting research, and fostering collaboration among clinicians, patient families, and professional organizations worldwide. The society supports research by providing Young Investigator Research Grants through the Patient Safety, Education, and Research Fund (PSERF). Educational efforts include the Questions of the Week, OpenAnesthesia Collaboration, and the SPA lecture series, all of which are open source and available online (Table 2). The SPA has developed patient safety resources for anesthesia clinicians taking care of children including the critical events checklist, Pedi Crisis App, position statements, newsletters, committees, and partnerships (Table 2).

SPA hosts subsocieties, sections, and partnerships (Table 3). sections include the Congenital Cardiac Anesthesia Society (CCAS), Society for Pediatric Pain Medicine (SPPM), Pediatric Anesthesia Leadership Council (PALC), and the Pediatric Anesthesia Program Director’s Association (PAPDA). Affiliates include the Pediatric Regional Anesthesia Network (PRAN), Women’s Empowerment and Leadership Initiative (WELI), Wake Up Safe, and SmartTots. Various committees are available for members to join based on individual interest (Table 4).

### 2.3. Multi-institution Collaborations

Pediatric anesthesia departments and divisions in the United States have created multi-institution collaborations to share data and resources regarding specific clinical problems. These are listed below:

- Pediatric Difficult Intubation Registry (PeDiR)
  PeDiR is dedicated to assessing, understanding, and improving the outcomes of children with difficult direct laryngoscopy (DDL) by facilitating benchmarking, quality improvement, and research. The group provides site-specific and aggregate data to members.
- PEdiatric Airway Registry (PEAR)
  Registry started by APAGBI to enter and track pediatric difficult airway management to inform practice, training, and education in pediatric anesthesia.
- Pediatric Anesthesia COVID Collaboration (PEACOC)
  Data gathering by members on COVID-19 (coronavirus or SARS-CoV-2 virus disease) prevalence, institutional practice, and outcomes in pediatric surgical patients during the pandemic that started in 2019.
- Pediatric Regional Anesthesia Network (PRAN)
  Collaborative institutions studying practice, risks, and complications of pediatric regional anesthesia. PRAN has a data repository for benchmarking, research, and quality improvement as well as a framework for organizing future large-scale multicenter studies.
- Pediatric Craniofacial Collaborative Group (PCCG)
  A multi-institutional group interested in pediatric craniofacial defect repair, especially craniosynostosis. They established the Pediatric Craniofacial Surgery Perioperative Registry (PCSPR) to study practices and outcomes in pediatric craniofacial surgery.
- Pediatric Heart Network (PHN)
  The National Heart, Lung, and Blood Institute (NHLBI) established the Pediatric Heart Network (PHN) as a multi-institution collaborative platform for clinical studies in patients with congenital heart disease and to provide data to guide practitioners.
- Wake Up Safe (WUS)
  Wake Up Safe collects data on perioperative anesthesia unexpected events to improve outcomes for children receiving anesthetic care as well as to educate members in improvement science.
- Strategies for Mitigating Anesthesia-Related neuroToxicity in Tots (SmartTots)
  Public–Private Partnership between International Anesthesia Research Society and the U.S. Food and Drug Administration. SmartTots identifies research questions, finds investigators to conduct studies, and funds pediatric anesthesia research with a focus on the neurotoxicity of sedatives.
- Task Force for Children’s Surgical Care
  Multidisciplinary group of perioperative care physicians formed to make recommendations to optimize pediatric surgical care. The American College of Surgeons, Children’s Hospital Association, and Task Force for Children’s Surgical Care, with input from related perioperative specialties, published standards for children’s surgical care [15].
- American College of Surgeons Children’s Surgery Verification (CSV) Quality Improvement Program
  Created to ensure optimal pediatric patient outcomes for patients receiving surgical care at verified healthcare facilities and offers institution verification to sites that meet the prescribed standards. This program is endorsed by the American Academy of Pediatrics, American Pediatric Surgical Association, and Society of Pediatric Anesthesiology [16].
- American Academy of Pediatrics—Section on Anesthesiology and Pain Medicine (SOA)
  Dedicated to educating pediatricians and other specialists in pediatric anesthesia and pain management. The Section serves as a forum for presentation of research projects, seminars, and conferences related to the perioperative care of children.
- North American Fetal Therapy Network (NAFTNET)
  Association of medical centers in the U.S. and Canada with expertise in fetal surgery and care for complex disorders of the fetus.

### 2.4. Collaborative Research

Large, recent collaborative multicenter studies dedicated to pediatric anesthesia were performed [17]. Two of these found no evidence of clinical anesthetic neurotoxicity. The General Anesthesia Spinal (GAS) study compared neurodevelopmental outcomes between two anesthetic techniques—general and regional—in infants and found no evidence that less than 1 h of sevoflurane increases risk of adverse neurodevelopmental outcomes at 2 years of age [18,19]. The Pediatric Anesthesia Neurodevelopmental Assessment (PANDA) prospectively assessed neuropsychological functions in a retrospective cohort of children and found that single anesthetic exposure did not have an increased risk of cognitive dysfunction [20]. The incidence of severe critical events in pediatric anesthesia (Anaesthesia PRactice In Children Observational Trial—APRICOT) study prospectively analyzed 30,874 children in 261 hospitals in Europe and reported perioperative critical events at 5.2%, with 10:10,000 anesthesia mortality [21]. The NEonate-Children sTudy of Anaesthesia pRactice IN Europe Epidemiology of Morbidity and Mortality in Neonatal Anaesthesia (NECTARINE) focused on neonates and included over 300 centers and more than 25,000 patients, with a focus on morbidity and mortality rates—data from this study are not yet published [22]. These studies showcase how conclusions may be best drawn by collaboration. This method should be repeated for other important pediatric anesthesia questions described in a parallel manuscript submitted by our group: “Hot Topics in Safety for Pediatric Anesthesia”.

### 2.5. International Pediatric Anesthesia Resources

Pediatric anesthesia resources are available online for international use. This includes the PedsAnesthesia.Net forum and the Optimal Resources for Children’s Surgery, which details pediatric surgical and anesthesia guidelines for primary health centers, first-, second-, and third-level hospitals, and national children’s hospitals [23]. The Safer Anesthesia from Education (SAFE) Pediatrics handbook is available at the WFSA website. Managing emergencies in pediatric anesthesia (MEPA) is a pediatric-focused international simulation training course offered in multiple countries with content and scenarios developed by the Royal College of Anesthetists U.K. [12,24]. 

Efforts to increase pediatric anesthesia capacity in low-resources settings are underway and include WFSA-sponsored pediatric anesthesia fellowships for low- and middle-income countries with 14 available in Asia, 11 in Africa, 12 in Latin America, 1 in Europe, and 2 in North America. The Safe Pediatric Anesthesia Network (SPAN) is a global academic exchange to discuss pediatric anesthetic and surgical themes. 

## 3. Discussion

Pediatric anesthesia has advanced substantially since it was first established as a subspecialty. Having anesthesiologists dedicated to the field has played a vital role in creating safer and better quality anesthetic care for our youngest and most vulnerable patients. The development of pediatric anesthesia as a subspecialty has subsequently led to the creation of multiple national and international societies. One of these societies formed was SPA. Since SPA was developed in 1986, there has been a flourishment in creating and developing various subsocieties, affiliations, committees, special interest groups, educational efforts, and patient safety resources. The multi-institutional national and international collaborations that are supported and endorsed by SPA provide the framework and encouragement for exchange of information, large data gathering, development of guidelines and consensus statements, and changes in practice that continue to move the subspecialty forward.

A perfect example of this is the PCCG. This special interest group began as a craniofacial data task force subcommittee of the SPA Bioinformatics group. Several interested SPA members first met in 2011 with the initial goal of determining what data and terminology should be used for craniofacial research. By the end of the meeting, the taskforce decided that a multicenter registry was needed in order to produce high-quality research. A pediatric anesthesiologist spearheaded the project and then recruited pediatric anesthesiologists at other children’s hospitals from across the country to participate. The PCCG was formed in 2012, and currently there are over 35 participating centers in the United States, with some additional international sites also contributing data. To date, there are over 7000 patients included in the PCSPR, and from this large multicenter data registry, important questions about this unique patient population have been able to be answered. With eight publications in major journals, and more on the way, the PCCG has accomplished everything from a benchmarking study to evaluating the safety of antifibrinolytics to creating prediction models for blood transfusion requirements in pediatric craniofacial surgery. This kind of work at this magnitude would not have been possible without the collaborative effort from many individuals representing their institutions. It also serves as an impressive example of how progress can be made in a relatively short amount of time with the right kind of support and motivation.

The research, education, and advocacy benefits of medical collaborations and societies are not without costs. Many organizations charge fees for membership, which can be difficult for low- and middle-income (LMIC) colleagues. However, organizations such as the SPA have ameliorated these costs with tiered rates for LMIC members. Traditionally, maximizing the benefits of membership also requires a significant investment of time and expense for travel to regular society meetings and conferences. However, recent technological advances in digital communication have greatly improved the accessibility and the ease of national and international collaborations. If trends in medical specialization continue, the opportunities for expanded specialty and subspecialty associations are likely to increase. Both costs and barriers to collaboration will be reduced as this process continues. Given past historical and technological advancements, continued improvement in perioperative pediatric patient safety is expected as pediatric anesthesiology moves forward.

## 4. Conclusions

Specialty societies and collaboration in pediatric anesthesia are crucial for continuous improvement in the care of children. They promote research, education, quality improvement, and advocacy at the local, national, and international level.

## Figures and Tables

**Table 1:** Pediatric anesthesia societies.

**International**
Subcommittee on Pediatric Anesthesia of the World Federation of Societies of Anaesthesiologists
**Americas**
Society for Pediatric Anesthesia
Canadian Pediatric Anesthesia Society
Sociedad Mexicana de Anesthesiologica Pediatrica (Mexico)
**Europe**
European Society for Pediatric Anesthesia
Association of Pediatric Anesthesia of Great Britain and Ireland
Association Des Anesthésistes Réanimateurs Pédiatriques d’Expression Française (France)
Belgian Association for Pediatric Anesthesiology
Società Italiana di Anestesia, Analgesia e Terapia Intensiva Pediatrica (Italy)
Schweizerische Gesellschaft für Kinderanästhesie (Switzerland)
Sectie Kinderanesthesie (The Netherlands)
Swedish Society for Pediatric Anesthesia and Intensive Care
**Asia**
Asian Society of Pediatric Anesthesiologists
Philippine Society for Pediatric Anesthesia
Japanese Society of Pediatric Anesthesia
Indian Association of Pediatric Anesthesiologists
Korean Society of Pediatric Anesthesiologists
Russian Pediatric Anesthesiologists and Reanimatologists Association
**Oceania**
Society for Pediatric Anesthesia in New Zealand and Australia
**Africa**
Pediatric Anesthesia Community of South Africa

**Table 2:** Society for Pediatric Anesthesia (SPA) educational efforts and patient safety resources.

Educational Efforts	Patient Safety Resources
Questions of the Week OpenAnesthesia Collaboration SPA Lecture Series	Critical Events Checklist Pedi Crisis Application Position Statements Newsletters Committees Partnerships

**Table 3:** SPA subsocieties, sections, partnerships/affiliates.

Sections	Affiliates/Partners
Congenital Cardiac Anesthesia Society (CCAS) Society for Pediatric Pain Medicine (SPPM) Pediatric Anesthesia Leadership Council (PALC) Pediatric Anesthesia Program Director’s Association (PAPDA)	Pediatric Regional Anesthesia Network (PRAN) Women’s Empowerment and Leadership Initiative (WELI) Wake Up Safe (WUS) SmartTots

**Table 4:** SPA committees and special interest groups (SIGs).

Committees	Special Interest Groups (SIGs)
Quality and Safety Committee SPA Global Committee Research Committee Communications Committee Education Committee Finance and Membership Committee Governance Committee Committee on Diversity, Equity, and Inclusion Committee on Public and Professional Affairs (COPPA)	Biomedical Informatics Blood Management Disaster Preparedness Fetal Anesthesia Pediatric Craniofacial Collaborative Group (PCCG) Pediatric Critical Care Medicine (PCCM) PeDiR-Airway Pediatric Difficult Intubation (PeDi) Registry Group Pediatric Liver and Intestinal Transplant (PLIT) Pediatric Perioperative Surgical Home Simulation Sustainability Well-being
